# Laparoscopic Sleeve Gastrectomy in a Patient With Situs Inversus: A Case Report

**DOI:** 10.7759/cureus.104267

**Published:** 2026-02-25

**Authors:** David Vu, Nigel Rajaretnam

**Affiliations:** 1 General Surgery, Austin Health, Melbourne, AUS

**Keywords:** bariatric surgery, laparscopic sleeve gastrectomy, mirror-image anatomy, obesity, situs inversus

## Abstract

Laparoscopic sleeve gastrectomy (LSG) is the most commonly performed bariatric surgery worldwide and is an effective treatment for obesity. Situs inversus (SI) is an uncommon congenital abnormality characterised by the anatomical location of organs within the body; it presents a rare technical challenge for bariatric surgeons due to the cognitive dissonance associated with operating on patients with mirror-image anatomy. Though even rarer, some of these patients may present with features of heterotaxy, which is defined by the abnormal arrangement of organs that comprises variants such as polysplenia. Considering the rarity of this condition, particularly in the context of bariatric surgery, our experience may provide valuable insight into the surgical approach for others who may encounter similar scenarios. We present a case of a 24-year-old female patient with SI presenting for elective LSG, with particular emphasis on the surgical technique that allowed us to successfully perform this procedure in the setting of reversed visceral anatomy. While no single approach can be universally recommended for SI, our described approach was successful and may assist surgeons who encounter these cases infrequently, particularly in centres without prior experience in mirror-image bariatric surgery.

## Introduction

Bariatric surgery has emerged as a durable tool to combat a rising global obesity epidemic [1]. In particular, LSG has become the most commonly performed bariatric procedure due to its technical simplicity, favourable safety profile, and ability to provide sustained weight loss [2]. This procedure also offers patients substantial metabolic benefits, including remission or improvement of metabolic syndromes, obstructive sleep apnoea, as well as a reduction in long-term cardiovascular risk, obesity-related malignancy incidence, and overall mortality [3-5].

SI is a rare congenital condition characterised by complete or partial mirror-image transposition of thoracic and abdominal organs, with an estimated incidence of ~0.01% [6]. It is frequently discovered incidentally, as most affected individuals remain asymptomatic [6]. SI, however, poses unique challenges in the surgical setting due to the cognitive dissonance associated with operating on a patient with altered anatomical orientation [7]. Alternatively, heterotaxy is defined by the abnormal arrangement of organs across the left-right axis, with left-sided isomerism (i.e., duplication of left-sided morphological structures) including variants such as polysplenia [8]. These patients have an increased risk of cardiac, respiratory, and renal abnormalities [9], though many cases still present without these heterotaxy syndromic features and are discovered incidentally [8]. Nonetheless, the coexistence of mirror-image anatomy with features of heterotaxy is even rarer than SI and poses additional challenges for the bariatric surgeon [8]. 

The coexistence of SI and morbid obesity requiring metabolic surgery is uncommon, and reports of bariatric surgery in this patient population remain limited to isolated case reports and small case series [7,10], with even fewer featuring patients with SI and left-sided isomerism [8]. As bariatric surgery continues to expand, awareness of anatomical variants and the technical strategies required to safely manage them is increasingly important. This case report contributes to the limited existing literature by describing the successful performance of LSG in a patient with SI and polysplenia.

## Case presentation

A 24-year-old female patient was referred for elective bariatric surgery. She measured 162 cm in height, weighed 111 kg, and had a body mass index of 42 kg/m². Relevant comorbidities included moderate obstructive sleep apnoea without current continuous positive airway pressure use and prediabetes. She was a non-smoker, a social drinker, and was receiving 15 mg tirzepatide weekly.

**Figure 1:**
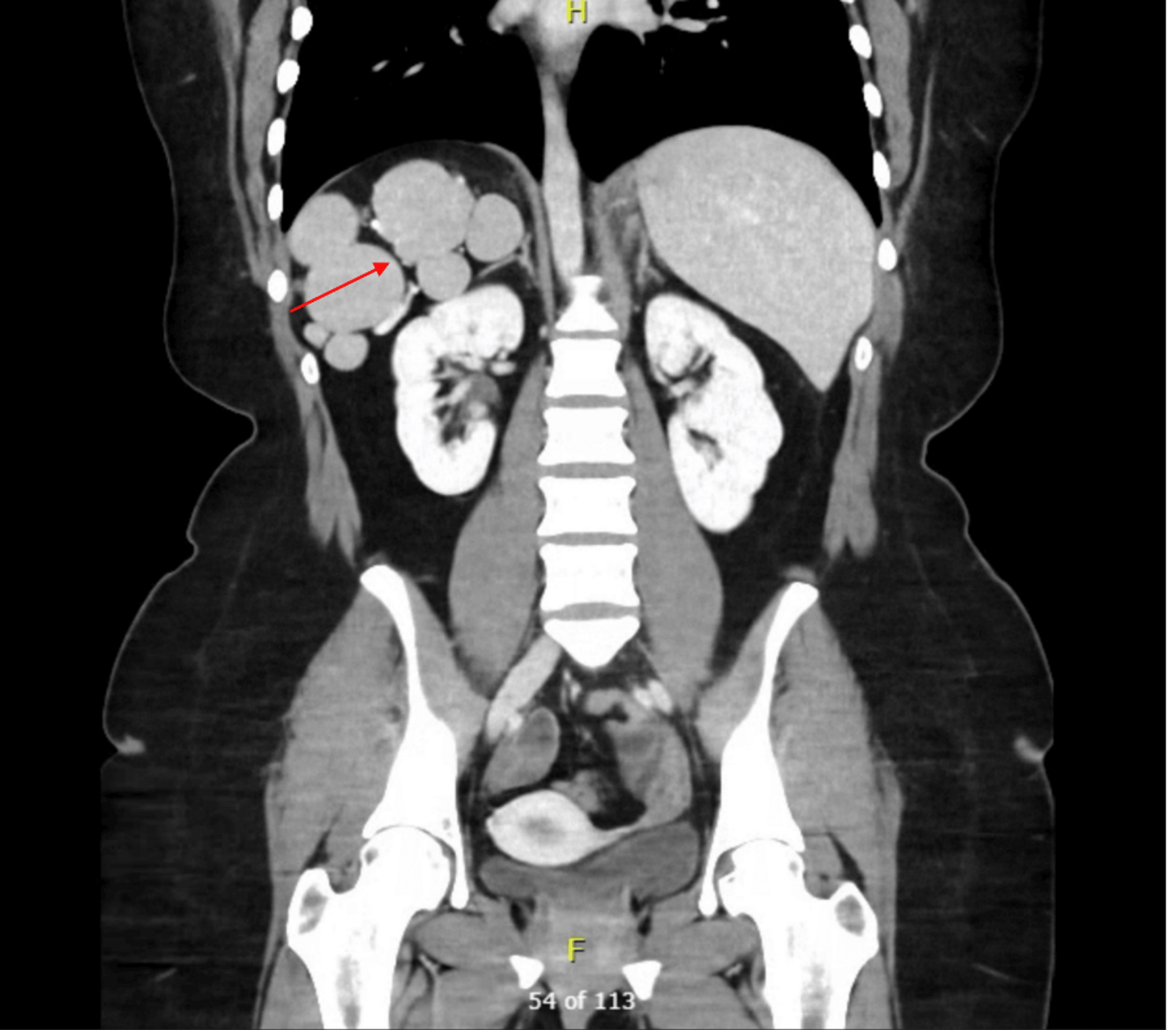
Computed tomography of the abdomen and pelvis with contrast Coronal section displaying left-sided liver, with the red arrow indicating polysplenia in the right upper quadrant.

**Figure 2:**
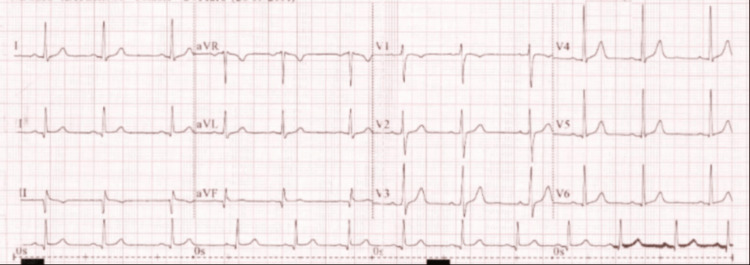
Electrocardiogram Normal electrocardiogram showing normal sinus rhythm.

In prior workup for a laparoscopic cholecystectomy, the patient received an ultrasound demonstrating a left-sided gallbladder, which raised suspicion for SI. During our workup for LSG, she received a computed tomography (CT) scan of the chest, abdomen, and pelvis (CTCAP), which demonstrated SI with dextrocardia, as well as polysplenia (Figure 1) and an atrophic pancreas. The patient had no history of respiratory, cardiac, or renal issues and no family history of developmental defects. A full preoperative assessment was also conducted, with normal physical exam, full blood count, coagulation profile, metabolic profile, thyroid function tests, haemoglobin A1c (5.4%, reference range 0-5.7%), and electrocardiogram (Figure 2). The patient was then cleared for surgery.

With the patient in low lithotomy position, the abdomen was accessed via a 12 mm supraumbilical optical trocar. Pneumoperitoneum was established, and two 12 mm ports were placed in the left upper quadrant and another 5 mm assisting port in the right lower quadrant. A Nathanson liver retractor (Mediflex Surgical Products, Inc., New York, United States) was placed via a small epigastric incision. Diagnostic laparoscopy revealed that all abdominal organs were in dextro-orientation (Figure 2).

**Figure 3:**
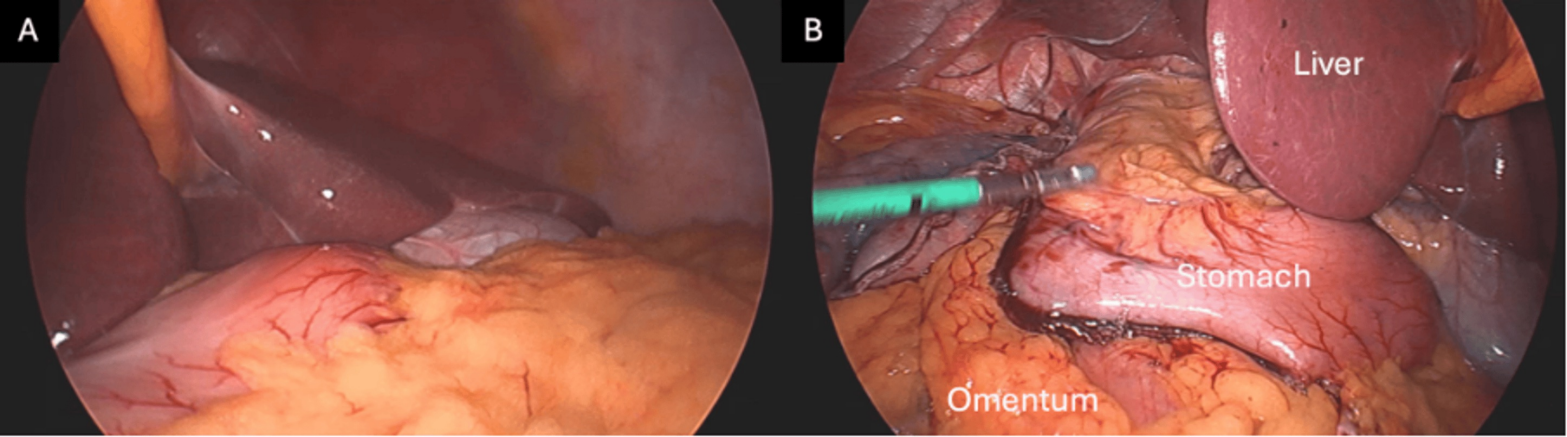
Laparoscopic gastric sleeve procedure A: initial laparoscopic view showing left-sided liver; B: laparoscopic view showing stomach (with staple line reinforcement), liver and omentum

The greater omentum was then dissected with LigaSure (Medtronic plc, Galway, Ireland), moving up the angle of His until the right crus was visualised. An Echelon Green and Blue 60 mm stapler (Ethicon, Inc., Raritan, New Jersey, United States) was used to resize the stomach with assistance of an intragastric 36Fr bougie, with stapling line reinforcement to reduce bleeding. Omentopexy was then performed with 2-0 prolene. The specimen and ports were removed, and the skin was closed with 3-0 monocryl. Total operative time was 62 minutes, with minimal estimated blood loss (20-30 mLs). The patient had an uncomplicated postoperative course, was able to resume oral intake with 100 mLs of free fluids four hours after surgery, and was able to commence mobilisation six hours postoperatively with minimal pain.

She was discharged 24 hours postoperatively with hospital-in-the-home services to monitor her oral intake. She had adequate oral fluid intake of 1L on 48 hours postoperatively. At the two-month clinical review, the patient's weight was measured at 92 kg, equivalent to approximately 17% total weight loss. 

## Discussion

This report describes a scenario that has only been reported anecdotally in previous literature. SI in bariatric surgery is a rare entity, and only a handful of case reports have described surgical techniques to contend with the anatomical challenge presented by these patients [10]; even fewer have specifically focused on LSG [11]. We present our own unique method of managing SI with polysplenia in a young patient undergoing LSG. Importantly, her 17% total weight loss at two-month clinical review is comparable to the ideal 10-year results of >20% reported in prior literature for non-SI patients, suggesting that mirror-image anatomy did not compromise weight-loss outcomes [2].

Preoperative assessment is particularly important in bariatric patients with SI to ensure preparedness of the surgical team. Cross-sectional imaging, typically with contrast-enhanced CT, is essential to confirm the extent of visceral transposition and prepare the surgeon prior to operating [11]. Our patient received a preoperative CTCAP, which confirmed SI and demonstrated polysplenia in the right upper quadrant and partial agenesis of the pancreas. SI with features of left-sided isomerism, including polysplenia, suggests an even rarer condition beyond isolated mirror-image anatomy that also overlaps with the heterotaxy spectrum [8]. Importantly, our patient had a normal preoperative electrocardiogram and laboratory tests, with SI and left-sided isomerism being discovered incidentally. She was thereby assessed to have low risk of heterotaxy syndromic features and was cleared by our anaesthetics team prior to surgery. Nonetheless, this feature further adds to the uniqueness and utility of our study, especially in the context of operative planning.

Prior literature has identified polysplenia as a potential source of complications in patients with SI due to an anomalous blood supply that is dissected during LSG [10,12]. Cross-sectional imaging may allow the surgeon to anticipate these additional anatomical variants as well as the source of potential postoperative complications if they arise. We thereby recommend a thorough review of this preoperative workup to obviate intraoperative disorientation, predict intraoperative challenges, and plan trocar placement and surgeon positioning to minimise ergonomic difficulties imposed by mirror-image anatomy [11,13]. Though not present in our patient, SI may also be associated with other congenital conditions, including primary ciliary dyskinesia and cardiopulmonary anomalies [6], which have important implications for anaesthetic planning [10]. Although SI itself does not contraindicate bariatric surgery, careful multidisciplinary evaluation is critical to achieving safe and reproducible outcomes in this rare patient population.

Careful consideration of port placement is vital for these patients, especially when considering the lack of established guidelines [7]. Previously, various approaches have been described in the literature. Pérez-Corzo et al. reported placement of a 12 mm supraumbilical camera port, with additional 12 mm ports in the right and left mid-clavicular lines, and a 5 mm port at the anterior axillary line for retraction [7]. Though favouring slightly different approaches, this mirrored configuration with symmetric distribution of ports has been utilised by other studies [14,15]. Regardless of specific technique, the decision regarding port placement is at the discretion of the surgical team, who must adapt their strategy according to the situation.

In contrast to many published SI cases, the present case utilised two 12 mm ports positioned ipsilaterally in the left upper quadrant, with a 5 mm assisting port in the right lower quadrant. The optical port was also placed on the patient’s right rectus muscle, in reverse to the usual position. This represents more lateralised port placement, concentrating instrumentation on the side of the reversed stomach to reduce instrument crowding and improve linear stapler alignment along the reversed greater curvature, particularly during proximal gastric mobilisation and dissection of the fundus. Concentrating larger working ports ipsilateral to the stomach may also decrease the need for awkward cross-handed movements, which have been noted as a challenge in mirror-image laparoscopic surgery [16]. Finally, although not necessary for our patient, the surgeon may decide to add more trocars [17] to ease ergonomic difficulty and reduce the technical challenges encountered in these patients.

Another difficulty in our case was performing gastric dissection and stapling via the non-dominant left hand, which increases technical complexity. This additional ergonomic strain was combated with careful attention, the trocar placement previously described, and flexible surgeon positioning, with occasional operating from the patient’s left side as required. Finally, a crucial takeaway from our study is the importance of always being aware of the dextro-orientation of the stomach and taking moments when necessary to overcome cognitive dissonance when operating with anomalous anatomy [17].

## Conclusions

SI, especially with features of heterotaxy, presents a rare but important technical challenge in bariatric surgery due to mirror-image anatomical variants and the absence of established operative guidelines. This case demonstrates that LSG can be performed safely with comparable weight-loss outcomes in patients with SI and polysplenia with thorough preoperative assessment and adapted, ergonomically driven operative planning. As the volume of bariatric surgery continues to rise, documenting varied and successful technical approaches may assist surgeons who encounter this uncommon anatomical variant, especially in centres without previous experience in mirror-image surgery. 
